# Venom of the Brazilian Spider *Sicarius ornatus* (Araneae, Sicariidae) Contains Active Sphingomyelinase D: Potential for Toxicity after Envenomation

**DOI:** 10.1371/journal.pntd.0002394

**Published:** 2013-08-22

**Authors:** Priscila Hess Lopes, Rogério Bertani, Rute M. Gonçalves-de-Andrade, Roberto H. Nagahama, Carmen W. van den Berg, Denise V. Tambourgi

**Affiliations:** 1 Immunochemistry Laboratory, Butantan Institute, São Paulo, Brazil; 2 Special Laboratory of Ecology and Evolution, Butantan Institute, São Paulo, Brazil; 3 Institute of Molecular and Experimental Medicine, School of Medicine, Cardiff, United Kingdom; Brasil Institute of Biomedical Sciences, Brazil

## Abstract

**Background:**

The spider family Sicariidae includes two genera, *Sicarius* and *Loxosceles*. Bites by *Sicarius* are uncommon in humans and, in Brazil, a single report is known of a 17-year old man bitten by a *Sicarius* species that developed a necrotic lesion similar to that caused by *Loxosceles*. Envenomation by *Loxosceles* spiders can result in dermonecrosis and severe ulceration. *Sicarius* and *Loxosceles* spider venoms share a common characteristic, *i.e.*, the presence of Sphingomyelinases D (SMase D). We have previously shown that *Loxosceles* SMase D is the enzyme responsible for the main pathological effects of the venom. Recently, it was demonstrated that *Sicarius* species from Africa, like *Loxosceles* spiders from the Americas, present high venom SMase D activity. However, despite the presence of SMase D like proteins in venoms of several New World *Sicarius* species, they had reduced or no detectable SMase D activity. In order to contribute to a better understanding about the toxicity of New World *Sicarius* venoms, the aim of this study was to characterize the toxic properties of male and female venoms from the Brazilian *Sicarius ornatus* spider and compare these with venoms from *Loxosceles* species of medical importance in Brazil.

**Methodology/Principal Findings:**

SDS-PAGE analysis showed variations in the composition of *Loxosceles* spp. and *Sicarius ornatus* venoms. Differences in the electrophoretic profiles of male and female venoms were also observed, indicating a possible intraspecific variation in the composition of the venom of *Sicarius* spider. The major component in all tested venoms had a Mr of 32–35 kDa, which was recognized by antiserum raised against *Loxosceles* SMases D. Moreover, male and female *Sicarius ornatus* spiders' venoms were able to hydrolyze sphingomyelin, thus showing an enzymatic activity similar to that determined for *Loxosceles* venoms. *Sicarius ornatus* venoms, as well as *Loxosceles* venoms, were able to render erythrocytes susceptible to lysis by autologous serum and to induce a significant loss of human keratinocyte cell viability; the female *Sicarius ornatus* venom was more efficient than male.

**Conclusion:**

We show here, for the first time, that the Brazilian *Sicarius ornatus* spider contains active Sphingomyelinase D and is able to cause haemolysis and keratinocyte cell death similar to the South American *Loxosceles* species, harmful effects that are associated with the presence of active SMases D. These results may suggest that envenomation by this *Sicarius* spider has the potential to cause similar pathological events as that caused by *Loxosceles* envenomation. Our results also suggest that, in addition to the interspecific differences, intraspecific variations in the venoms composition may play a role in the toxic potential of the New World *Sicarius* venoms species.

## Author Summary

The spider family Sicariidae includes two genera, *Sicarius* and *Loxosceles*. These spiders' venoms share a common characteristic, *i.e.*, the presence of Sphingomyelinases D (SMase D). This toxin is the main component responsible for the local and systemic effects observed in loxoscelism. In the present study, we have investigated the toxic potential of male and female Brazilian *Sicarius ornatus* spider venoms and compared these with the venoms from *Loxosceles* species of medical importance in Brazil. We show here that Brazilian *Sicarius ornatus* venom is endowed with all toxic *in vitro* and *ex vivo* biological properties ascribed to the venoms from *Loxosceles* species, including the abilities to hydrolyze sphingomyelin and to induce keratinocyte cell death and complement dependent haemolysis, detrimental effects that were positively associated with the presence of active SMases D and with *in vivo* pathologies. Therefore, the venom of *Sicarius ornatus* spider can potentially lead to a similar pathology as that observed for *Loxosceles* envenomation.

## Introduction

**Figure 1:**
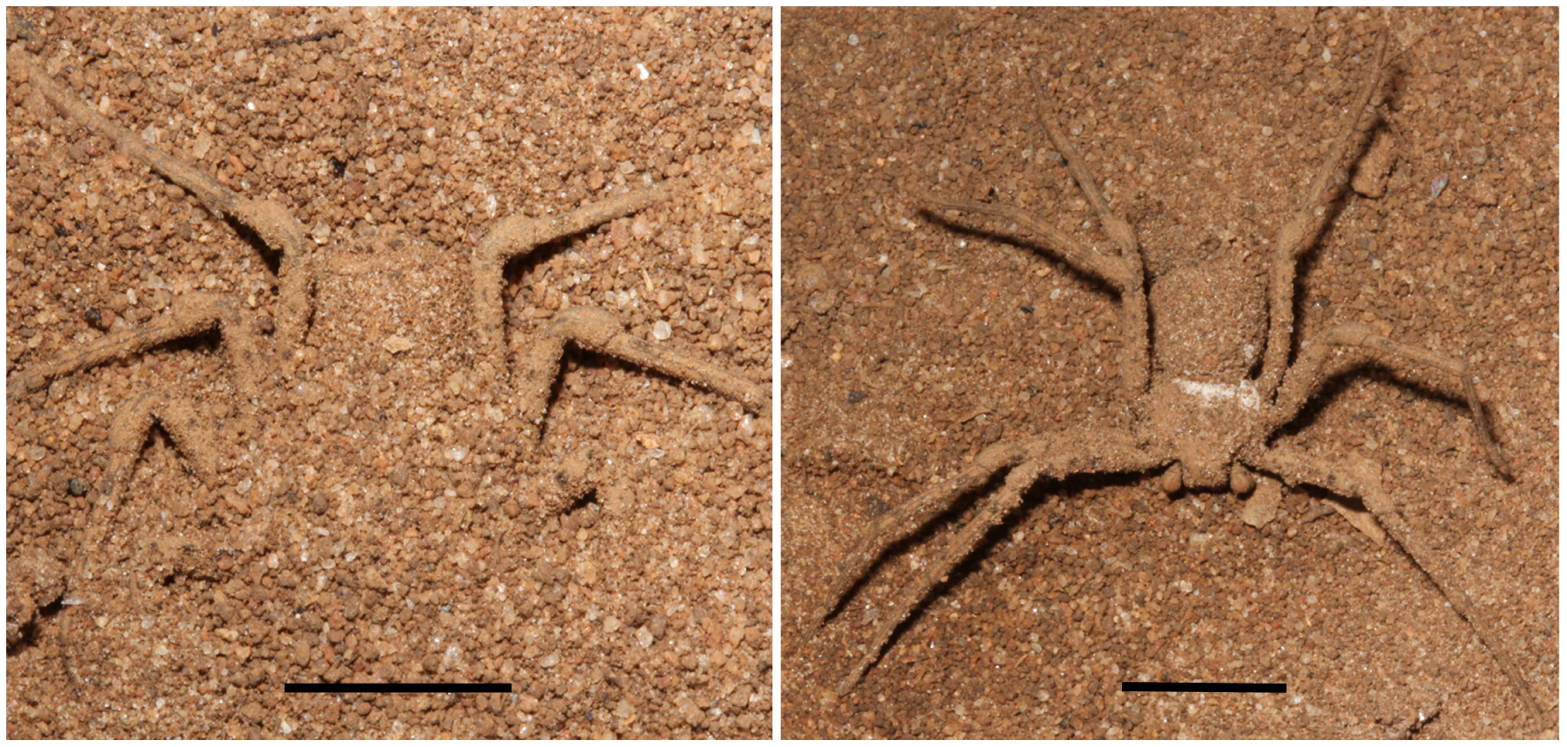
*Sicarius ornatus* male. Adult *Sicarius ornatus* spider collected in Elisio Medrado, State of Bahia, Brazil. Left: specimen partially buried into the sand; Right: specimen with its body characteristically incrusted with sand grains. Scale bar – 10 mm.

The spider family Sicariidae includes two genera, *Sicarius* and *Loxosceles*. *Sicarius* species (six-eyed crab spiders, six-eyed sand spiders) live in dry forests and deserts throughout Southern Africa, South America and Central America. The genus *Sicarius* is composed of robust flattened spiders, 9–19 mm long and a leg span of about 5 cm. The legs are laterally placed, resembling a crab, hence the common name. These spiders are found buried in soil layers, where they live and wait to trap their prey (Figure 1). They feed on passing insects, rapidly emerging from the sand when disturbed. During self-burial, soil particles can adhere to their specialized sethae (hairs), which cover their bodies, changing their natural coloration to the color of the environment [1], [2].

Bites by *Sicarius* are uncommon in humans. In Brazil, a single report is known of a 17-year old man bitten by a *Sicarius* species who developed necrotic lesion similar to that caused by *Loxosceles* [3]. Yet, several reports have shown that African *Sicarius* spider venoms are lethal to rabbits and are also able to cause dermonecrotic lesions in humans and in experimental animals [4]–[9].

The genus *Loxosceles* includes spiders of small dimensions, with a body length of about 10 mm and leg span of 3 cm, relatively fine long legs and six eyes organized in a characteristic pattern of three dyads in the shape of a U. Known popularly as brown spider in South America and as recluse spider in North America, the specimens have a dark or light brown violin-shaped mark on its carapace. *Loxosceles* spiders are nocturnal and prefer dry, dark and quiet places, and live under wood and rocks, under the bark of trees and in caves. They adapt very well to domiciliary conditions, hiding behind pictures, in furniture, in clothes and shoes, always protected from direct light [10]–[12].

Envenomation by *Loxosceles* spiders, considered one of the four dangerous forms of araneism [13], is a serious public health hazard in North and South America. Although systemic reactions such as shock, haemolysis, renal insufficiency and disseminated intravascular coagulation are rare, small areas of erythema often leading to larger areas of ulceration and necrosis are frequently observed [14], [15]. At least three different synanthropic *Loxosceles* species of medical importance are known in Brazil (*L. intermedia*, *L. gaucho*, *L. laeta*) and more than 5000 cases of envenomation by these spiders are reported each year.

*Sicarius* and *Loxosceles* spider venoms share a common characteristic, *i.e.*, the presence of Sphingomyelinases D (SMase D) [16]–[17]. Numerous studies have demonstrated that SMase D present in the venoms of *Loxosceles* spiders is the main component responsible for the local and systemic effects observed in loxoscelism [18]–[26]. SMases D hydrolyze sphingomyelin resulting in the formation of ceramide-1-phosphate and choline [18], [19], [21] and, in the presence of Mg^2+^, are able to catalyze the release of choline from lysophosphatidylcholine [27].

Recently, it was demonstrated that *Sicarius* species from Africa, like *Loxosceles* spiders from the Americas, present high venom SMase D activity. However, despite the presence of SMase D like proteins in venoms of several New World *Sicarius* species tested, as the ones from Argentina (*S. terrosus*, *S. rupestris*, *S. patagonicus*), Peru (*S. peruensis*) and Costa Rica (*S. rugosus*), these venoms had reduced or not detectable SMase D activity [17]. In order to contribute to a better understanding about the toxicity of New World *Sicarius* venoms, the aim of this study was to characterize the biochemical and biological properties of male and female venoms from a Brazilian *Sicarius* species, *Sicarius ornatus*, and compare these with venoms from *Loxosceles* species of medical importance in Brazil.

## Materials and Methods

### Chemicals, reagents and buffers

Tween 20, bovine serum albumin (BSA), paraformaldehyde, 3-(4,5 dimethylthiazol-2yl)-2,5 diphenyltetrazolium bromide (MTT), sphingomyelin (SM), choline oxidase, horseradish peroxidase (HRPO) and 3-(4-hydroxy-phenyl) propionic acid were purchased from Sigma Co. (St. Louis, MO, USA). 5-bromo-4-chloro-3-indolyl-phosphate (BCIP), nitroblue tetrazolium (NBT) and goat anti-rabbit IgG-alkaline phosphatase (GAR/IgG-AP) were from Promega Corp. (Madison, WI, USA). Rabbit anti-mouse IgG-FITC (RAM-FITC) and goat anti-rabbit IgG-FITC (GAR-FITC) were from Amersham Pharmacia Biotech (Buckinghamshire, UK). Monoclonal antibody against GPC (Bric4, extracellular epitope aa 16–23) was from IBGRL (Bristol, UK). Rabbit serum against SMases D from *L. intermedia* venom was obtained as previously described [20]. Buffers were: veronal-buffered saline (VBS^2+^), pH 7.4: 10 mM NaBarbitone, 0.15 mM CaCl_2_ and 0.5 mM MgCl_2_; phosphate-buffered saline (PBS), pH 7.2: 10 mM NaPhosphate, 150 mM NaCl; fluorescence activated cell sorter (FACS) buffer, containing PBS, 1% BSA, 0·01% sodium azide. HEPES-buffered saline (HBS), pH 7.4: 10 mM Hepes: 140 mM NaCl, 5 mM KCl, 1 mM CaCl_2_, 1 mM MgCl_2._

### Spiders and venoms

Adult male (n = 3) and female (n = 5) *Sicarius ornat*us spiders (Figure 1) were collected in Elisio Medrado, State of Bahia, RPPN Jequitiba (capture and maintenance licenses from IBAMA, Brazil, number 13676-1). Eleven voucher specimens were deposited at Arachnology Laboratory, Museu Nacional do Rio de Janeiro (MNRJ) under accession numbers 06479 to 06485. Adult females of *Loxosceles laeta*, *L. gaucho* and *L. intermedia* were provided by Immunochemistry Laboratory, Butantan Institute, Brazil (capture and maintenance licenses from IBMA, Brazil, number 11971-2). We considered as adults spiders those specimens with fully developed palpal copulatory organs (males) or with an epigastric furrow with a clearly visible opening of the oviduct (females). The venoms were obtained by electrostimulation by the method of Bucherl [28] with slight modifications. Briefly, 15–20 V electrical stimuli were repeatedly applied to the spider sternum and the venom drops were collected with a micropipette in PBS, aliquoted and stored at −20°C. The protein content of the samples was evaluated using the BCA Protein Assay Kit (Pierce Biotechnology, MA, USA).

### Electrophoresis and Western blotting

Venom samples (10 µg of protein) from *Sicarius ornatus* (male and female) or *Loxosceles* spp. (female) were solubilised in non-reducing sample buffer, run on 12% SDS-PAGE [29] and silver stained. Alternatively, gels were blotted onto nitrocellulose [30]. After transfer, the membranes were blocked with PBS containing 5% BSA and incubated with rabbit serum anti-native SMases D from *L. intermedia* venom (diluted 1∶250) for 1 h at room temperature. Membranes were washed three times with PBS/0.05% Tween 20 for 5 min each wash, and incubated with GAR/IgG-AP (1/7500) in PBS/1% BSA for 1 h at room temperature. After washing three times with PBS/0.05% Tween 20, for 5 min each wash, blots were developed using NBT/BCIP according to the manufacturer's instructions (Promega).

### Enzymatic activity

The SMase D activity of the venoms was estimated by determining the choline liberated from lipid substrates, using a fluorimetric assay [31]. Briefly, sphingomyelin (SM – 50 µM) was diluted in 1 mL HEPES-buffered saline (HBS), samples of *Sicarius ornatus* or *Loxosceles* spp. venoms (10 µg of protein) were added and the reaction was developed for 30 min at 37°C. After incubation, a mixture consisting of 1 unit choline oxidase/mL, 0.06 units of horseradish peroxidase/mL and 50 µM of 3-(4-hydroxy-phenyl) propionic acid in HBS was added and incubated for 10 min. The choline liberated was oxidized to betaine and H_2_O_2_ and this product determined by fluorimetry at λem = 405 nm and λex = 320 nm, using 96-well microtiter plates, in a spectrofluorimeter (Perkin-Elmer, USA).

### Normal human serum and erythrocytes

Human blood was obtained from healthy donors who knew the objectives of the study and signed the corresponding informed consent form approved by the ethics committee (CAAE: 07039213.3.0000.5467). Blood samples were collected without anticoagulant and allowed to clot for 4 hours at 4°C. After centrifugation, normal human serum (NHS) was collected and stored at −80°C. Blood samples drawn to obtain erythrocytes (E) for subsequent use as target cells were collected in anticoagulant (Alsever's old solution: 114 mM citrate, 27 mM glucose, 72 mM NaCl, pH 6.1).

### Treatment of erythrocytes with venoms

Human erythrocytes were washed and resuspended at 2% in VBS^2+^ and incubated with different concentrations of the venoms for 1 h at 37°C. Control samples were incubated with VBS^2+^. The cells were washed, resuspended to the original volume in VBS^2+^ and analysed in a haemolysis assay as described [20] or prepared for flow cytometry.

### Flow cytometry

Samples of human erythrocytes (25 µL) were incubated for 30 min with 25 µL of primary or control antibodies (1–10 µg/mL) in FACS buffer. After washing, cells were incubated with the appropriate FITC-labelled secondary antibodies for 30 min. The cells were washed and fixed in FACS buffer containing 1% paraformaldehyde and analysed by flow cytometry (FACScalibur, Becton Dickinson, California, USA).

### Human keratinocytes cultures

Human keratinocytes (cell line HaCaT) were maintained in DMEM (Gibco-BRL, Gaithersburg, MD, USA), supplemented with 10% (vol/vol) heat-inactivated (56°C, 30 min) foetal bovine serum (FBS; Cultilab, São Paulo, Brazil), 100 IU of penicillin/mL, and 100 IU of streptomycin/mL at 37°C in humidified air with 5% CO_2_.

### Viability assay

HaCaT cells were subcultured in 96-well plates (5×10^4^cells/well). Cells at 50%–70% confluence were maintained overnight in DMEM without FBS, followed by incubation with the venoms (10 µg of protein). DMEM without FBS was used as the control. After 48 and 72 hours, the viability of the cultures was tested by the MTT [32]. Supernatants of each sample (100 µL) were collected and mixed with 100 µL of water and the absorbance was measured in a spectrophotometer (Multiskan-EX, Labsystems, Helsinki, Finland) at 540 and 620 nm. The relative cell viability was calculated as: [(Sample OD_(540–620 nm)_ – Background control OD_(540–620 nm)_)/(Control OD_(540–620 nm)_ – Background OD _(540–620 nm)_)]×100.

### Statistical analysis

Data were analyzed statistically by one way ANOVA and Tukey test. A *P*-value<0.05 was considered significant.

## Results

### Determination of the protein concentration of *Sicarius ornatus* and *Loxosceles* venoms

**Figure 2:**
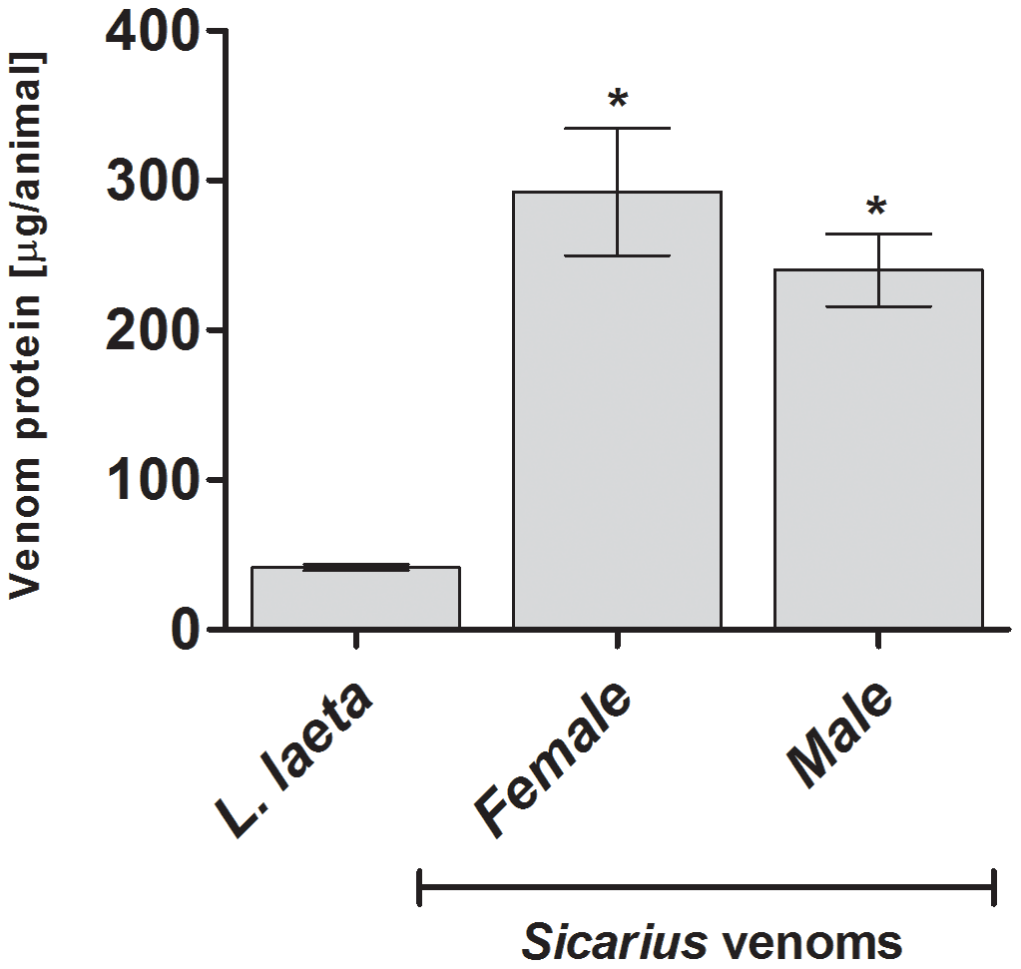
Protein content of *Sicarius ornatus* and *Loxosceles* venoms. The protein content of *Loxosceles laeta* females (n = 68), female (n = 5) and male (n = 3) adult *Sicarius ornatus* spiders venoms samples were determined using the BCA colorimetric method. Results are expressed as mean ± SD. (*) Significant difference (*P*<0.05) from *L. laeta* venom.

Figure 2 shows that the venom of male and female *Sicarius ornatus* spiders contain similar amounts of protein. Comparison analysis showed that male and female *Sicarius ornatus* venoms contain significant higher protein concentrations than *L. laeta* female venom.

### Immunochemical characterization of the *Sicarius* and *Loxosceles* venoms

**Figure 3:**
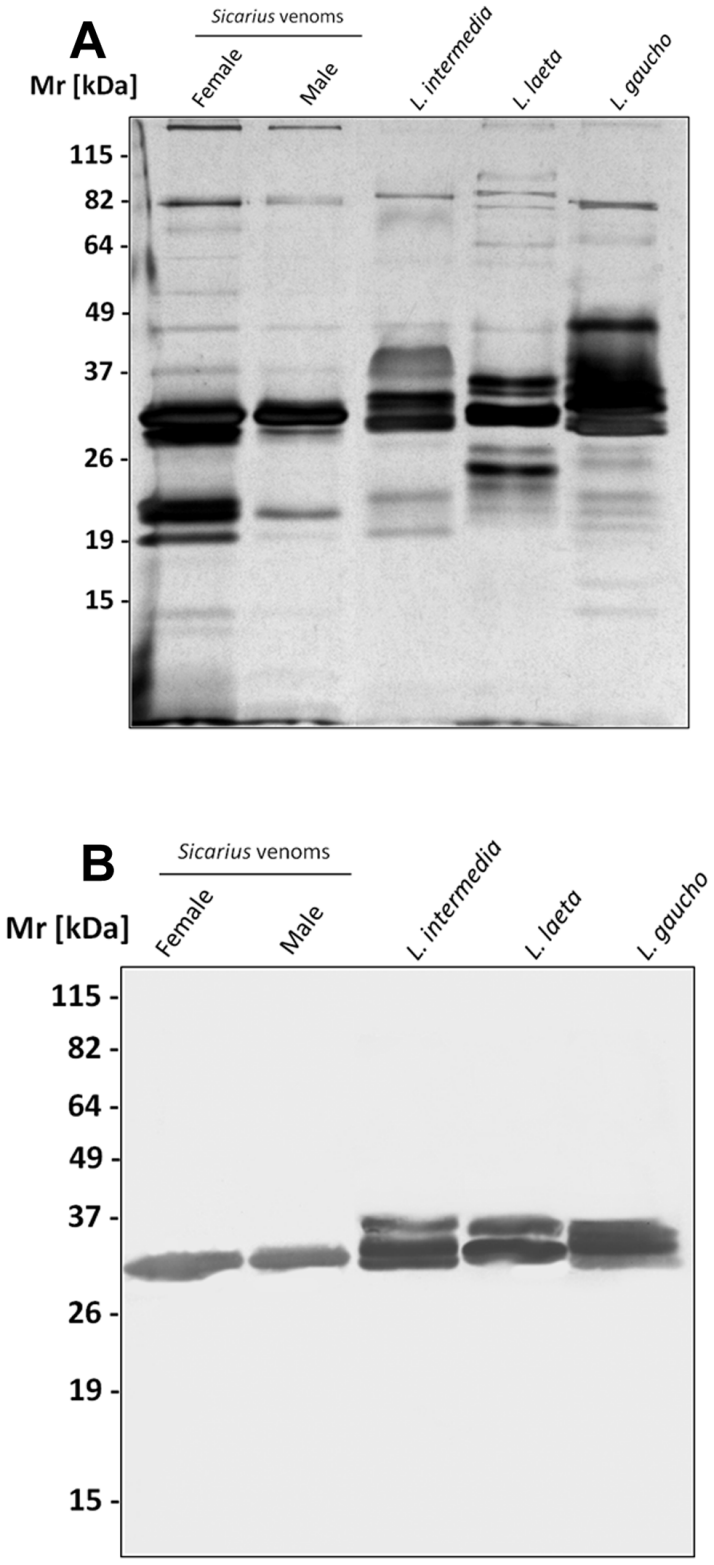
Eletrophoretic analysis of *Sicariu*s *ornatus* and *Loxosceles* venoms. Samples of the venoms (10 µg of protein) were subjected to electrophoresis on a 12% SDS-PAGE gel under non-reducing conditions, stained with silver [**A**] or western blotted [**B**]. Blot was probed with polyclonal serum against SMases D from *L. intermedia* diluted 1∶250, followed by anti-rabbit IgG/AP conjugate (1∶7.500) and the reaction developed using NBT/BCIP. Lane 1: *Sicarius ornatus* female venom; Lane 2: *Sicarius ornatus* male venom; Lane 3: *L. intermedia* venom; Lane 4: *L. laeta* venom; Lane 5: *L. gaucho* venom.

Comparative analysis of the spider venoms, by SDS-PAGE followed by silver staining, revealed differences in the number and intensity of bands of venoms from male and female *Sicarius ornatus* spiders and also from *Loxosceles* species, however, all venoms showed a major band with Mr of 32–35 kDa, which corresponds, in *Loxosceles* venoms, to the main toxic component, *i.e*, the SMase D (Figure 3A). In order to assess the identity of this band and analyze the inter- and intra-species cross-reactivities, polyclonal antiserum raised against a pool of purified SMases D from *L. intermedia* was used in western blot. Figure 3B shows that this antiserum strongly recognized the SMases D present in the venoms from *Loxosceles intermedia, L. laeta and L. gaucho* and also reacted with a band of similar Mr of approximately, 33 kDa in the *Sicarius ornatus* spider male and female venoms, suggesting that this band also corresponds to a sphingomyelinase D.

### Brazilian *Sicarius ornatus* spider venoms contain SMase D activity

**Figure 4:**
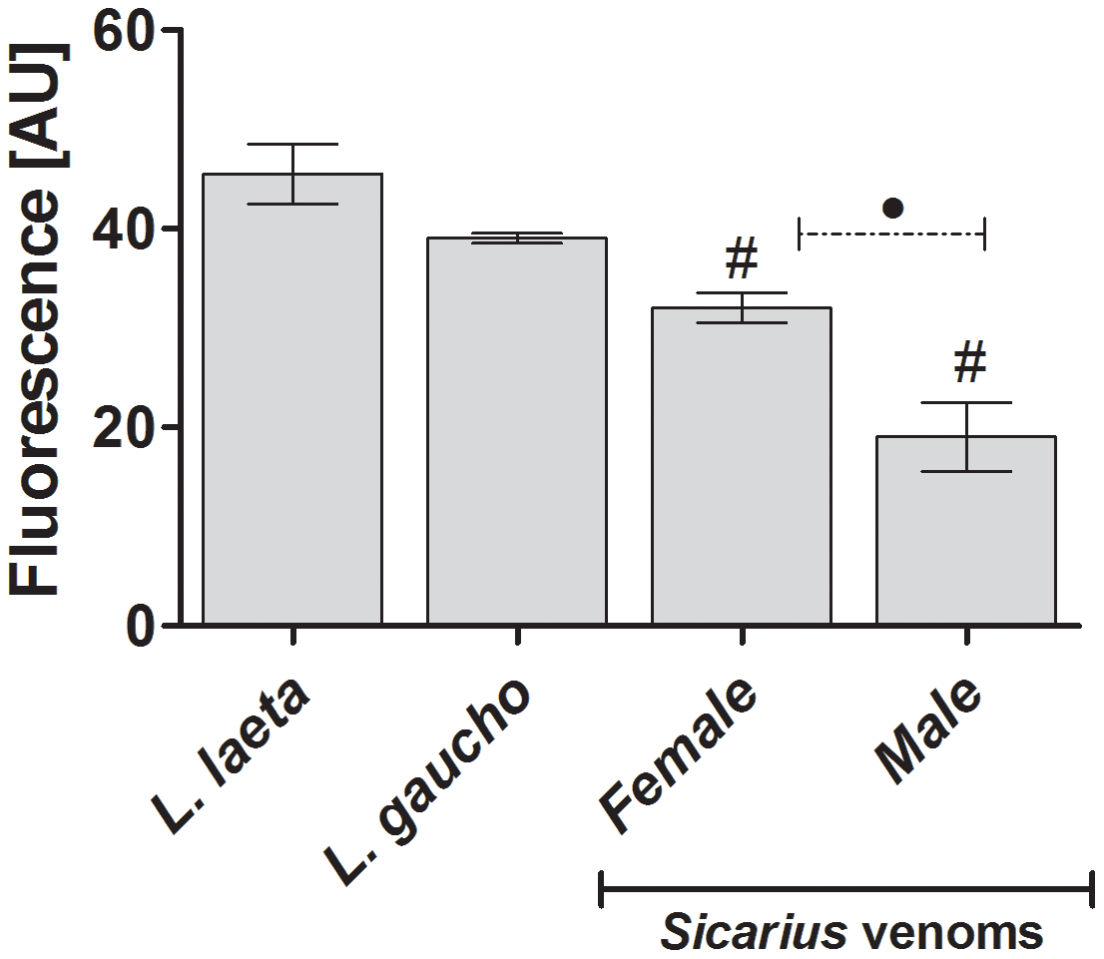
Sphingomyelinase activity of *Sicarius* venoms. Sphingomyelin (50 µM) was incubated with buffer or with *Sicarius ornatus* or *Loxosceles* spp. venoms. After 20 min at 37°C, the formed choline was oxidized to betaine and determined fluorimetrically. Results are representative for three separate experiments and expressed as mean ± SD of duplicates. The variation among experiments was around 10%. (#) Significant differences from *Loxosceles* spp. venoms (*P*<0.05); (•) Significant difference between male and female *Sicarius ornatus* venoms.

Figure 4 shows that all tested venoms, including from the *Sicarius ornatus* spider, were able to hydrolyze sphingomyelin. Comparative analysis revealed significant differences in the sphingomyelinase activity of venoms from *Loxosceles* species and *Sicarius ornatus* However, *Loxosceles* spp. and *Sicarius ornatus* female venoms exhibited a more potent sphingomyelinase activity than *Sicarius ornatus* male venoms (*P*<0.005).

### *Sicarius ornatus* venoms induce hemolysis

**Figure 5:**
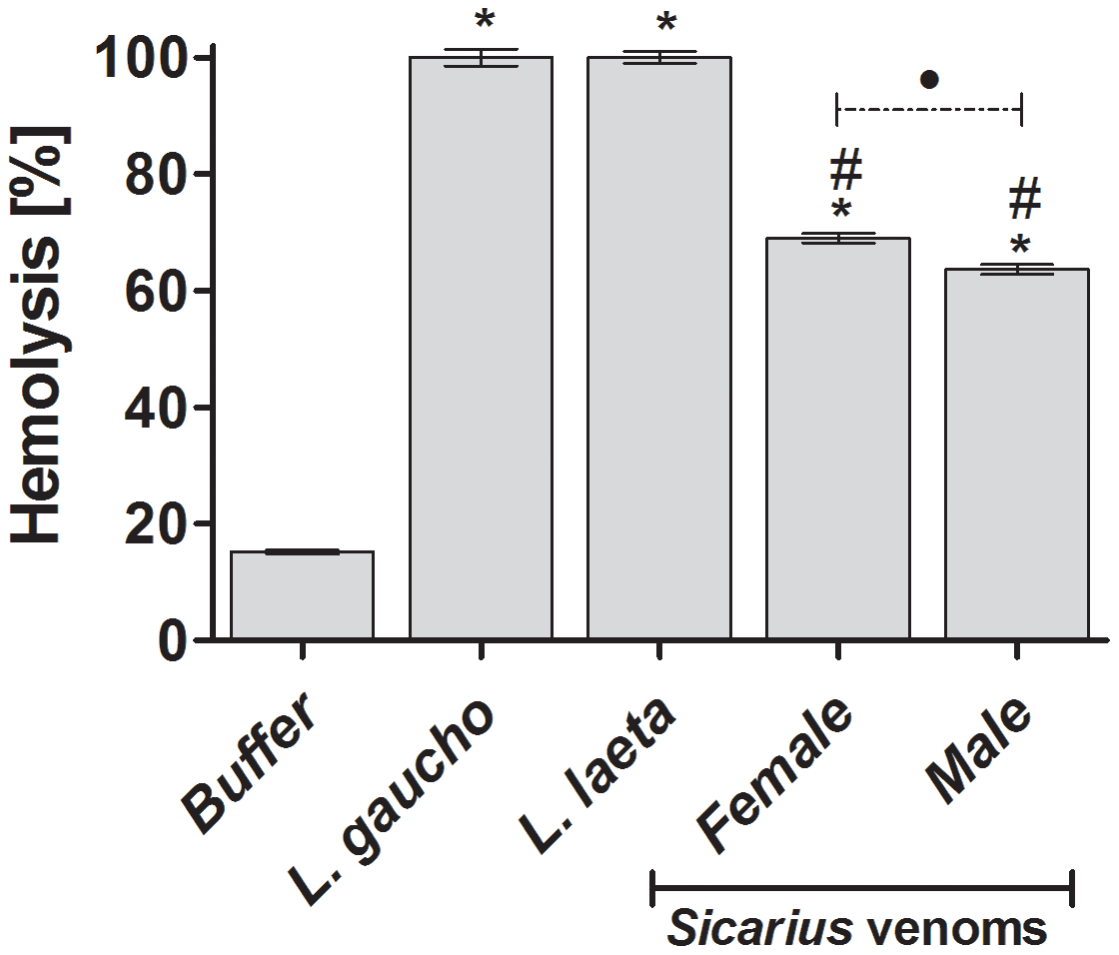
Hemolysis dependent of complement system. Human Erythrocytes, pre-treated with VBS^2+^ or with *Sicarius ornatus* or *Loxosceles* spp. venoms, were incubated with autologous normal human serum. After incubation for 1 h at 37°C, unlysed cells were spun down; the absorbance of the supernatants was measured at 414 nm and expressed as percentage of lysis. Results are representative for three different experiments and expressed as mean ± SD of duplicates. The variation among experiments was around 10%. (*) Significant differences (*P*<0.05) from control; (#) Significant differences from *Loxosceles* spp. venoms (*P*<0.05); (•) Significant difference between female and male *Sicarius ornatus* venoms (*P*<0.05).

To compare the spiders' venoms capability of inducing complement-dependent haemolysis, human erythrocytes were incubated *Sicarius ornatus* venoms or *Loxosceles* spp. venoms and incubated with normal human serum as a source of complement. Figure 5 shows that male and female venoms from *Sicarius ornatus* spiders, as well as from *Loxosceles*, were able to render human erythrocytes susceptible to lysis by autologous serum. A more potent complement-dependent haemolytic inducing activity was detected in *Loxosceles* spp. venoms (*P*<0.005).

**Figure 6:**
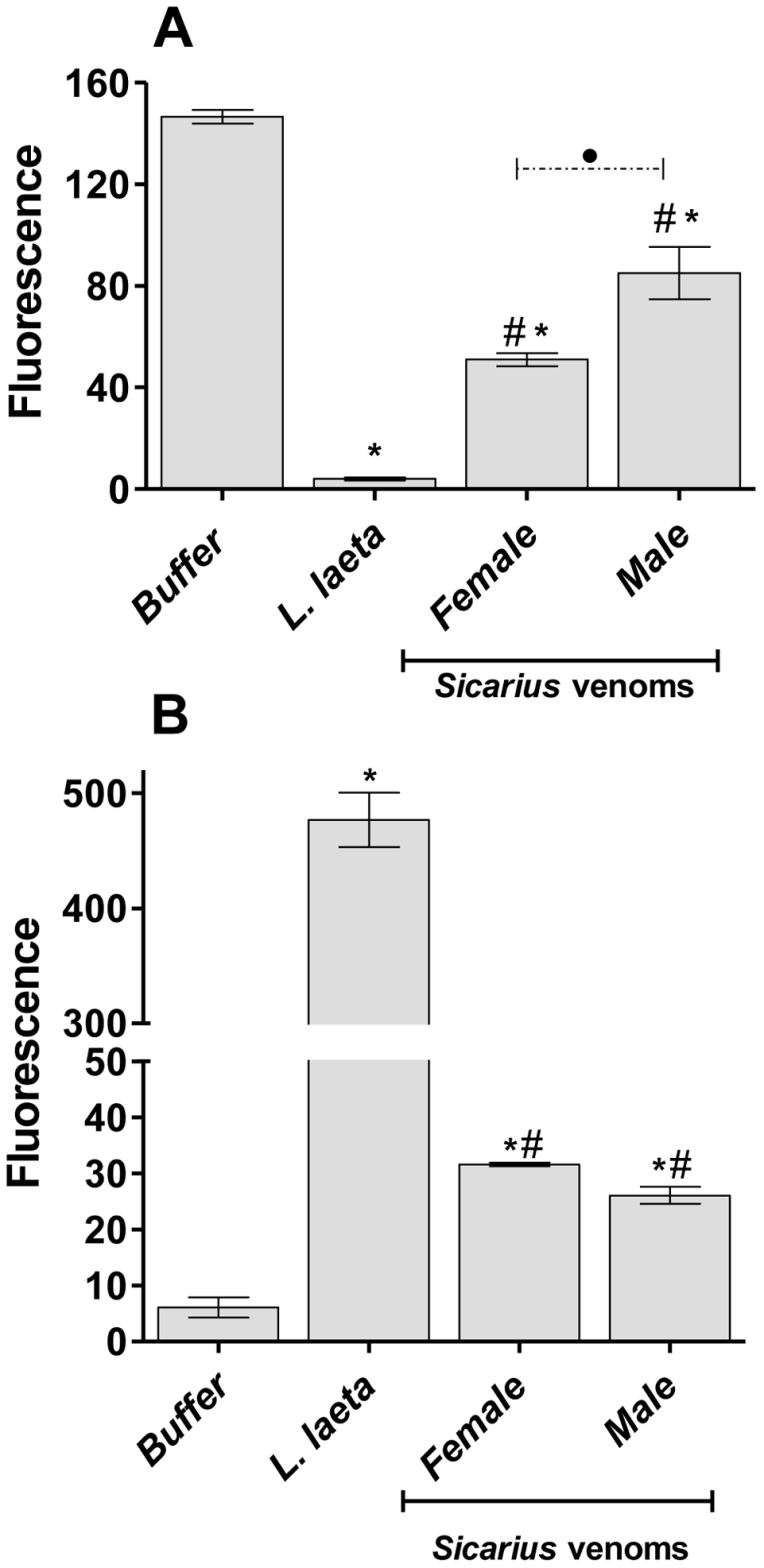
*Loxosceles* and *Sicarius ornatus* venom SMases D incorporate into human erythrocytes and cause loss of glycophorin C expression. Erythrocytes were treated with venoms from *Sicarius ornatus* or *Loxosceles* or with VBS^2+^ buffer (control) and analyzed for the expression GPC by flow cytometry [A]. The ability of the toxins to insert into the erythrocyte surface was analyzed using a monospecific polyclonal rabbit serum against *Loxosceles intermedia* SMases D [B]. Results are representative for three different experiments and expressed as median of fluorescence of duplicates ± SD. The variation among experiments was around 10%. (*) Significant differences (*P*<0.05) from control; (#) Significant differences from *L. laeta* venom (*P*<0.05); (•) Significant difference between female and male *Sicarius* venoms (*P*<0.05).

We have previously shown that removal of glycophorins (GPs) is partially responsible for the increased complement-susceptibility of erythrocytes treated with *Loxosceles* venoms. To investigate if *Sicarius ornatus* venoms induced a similar effect, human erythrocytes were incubated with *Sicarius ornatus* or *Loxosceles* venoms or buffer and analyzed for the expression of glycophorin C (GPC) by flow cytometry. A significant reduction in binding of anti-GPC antibodies was observed after treatment of erythrocytes with all venoms (#Figure 6A). Statistically significant differences (*P*<0.005) were detected between *Loxosceles* and *Sicarius ornatus* venoms and also between *Sicarius ornatus* genders, the female venom being more potent inducer of removal of GPs. The disappearance of GPs epitopes, induced by both *Sicarius ornatus* and *Loxosceles* venoms, was associated with the binding of the SMAses D to the erythrocyte cell surface as detected by anti-*Loxosceles* SMAse D anti-serum (Figure 6B). Nonetheless, the stronger reduction in the expression of GPs in *Loxosceles* venom treated cells, as compared with male and female *Sicarius ornatus* venoms, correlates positively with the higher SMase D cell binding and hemolytic complement-dependent inducing capabilities exhibited by the former venom.

### *Sicarius ornatus* venom reduces cell viability

**Figure 7:**
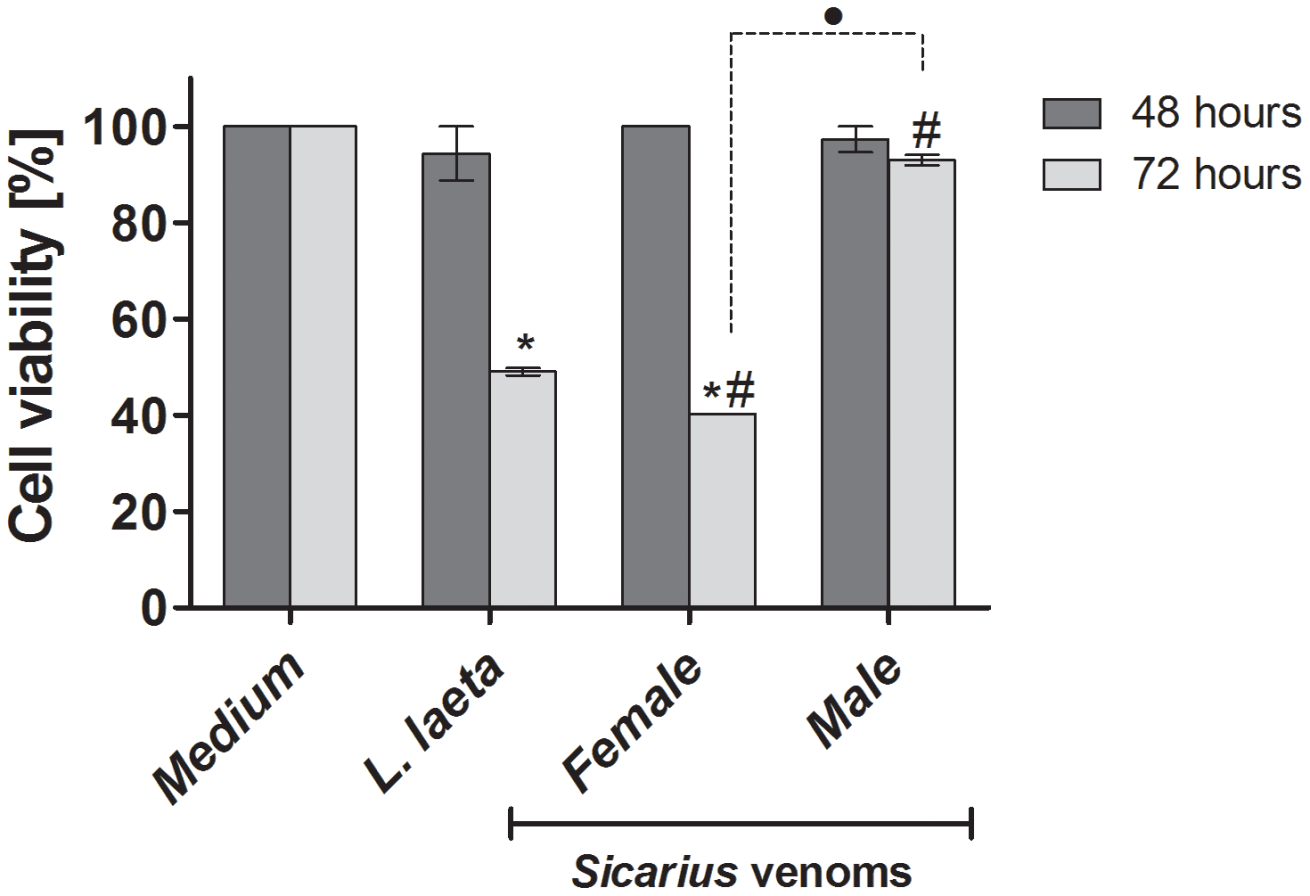
Effect of venoms on human keratinocytes viability. HaCaT cells (5×10^4^ cells/well) were cultured in 96-well plates with DMEM without FBS for 24 hours followed by incubation with venoms (10 µg of protein). After 48 h and 72 h, the viability was tested by the MTT method and the readings taken at wavelengths of 540–620 nm. Data are expressed as mean ± SD of duplicates. (*) Significant differences (*P*<0.05) from control; (#) Significant differences from *L. laeta* venom (*P*<0.05); (•) Significant difference between female and male *Sicarius ornatus* venoms (*P*<0.05).

Envenomation by *Loxosceles* spiders is a well-documented cause of necrotic skin lesions in humans. Using HaCaT cultures, a human keratinocyte cell line, as an *in vitro* model for cutaneous loxoscelism, we have shown that *Loxosceles* spider venom and its SMase D induce apoptosis in human keratinocytes. In order to analyze if the same toxic effect could be induced by *Sicarius ornatus* venoms, HaCaT cells were incubated with male or female *Sicarius ornatus* venoms or *L. laeta* venom during 48 or 72 h and the cell viability was analyzed by the MTT method. Figure 7 shows that both female *Loxosceles* and *Sicarius ornatus* venoms were able to induce a significant loss of cell viability after 72 h of incubation; the female *Sicarius ornatus* venom was more efficient in provoking the loss of cell viability than male *Sicarius ornatus* and female *Loxosceles* venoms.

## Discussion

In the present study, we have investigated the toxic potential of venoms from male and female Brazilian *Sicarius ornatus* spider, collected in Elisio Medrado, Bahia, Brazil and compared them with the venoms from *Loxosceles* species of medical importance in Brazil. We show here that this Brazilian *Sicarius ornatus* venom is endowed with all toxic biological properties ascribed to the venoms from *Loxosceles* species, including the abilities to hydrolyze sphingomyelin, to induce keratinocyte cell death and complement dependent haemolysis.

SDS-PAGE analysis showed variations in the composition of *Loxosceles* spp. and *Sicarius ornatus* venoms. Differences in the electrophoretic profiles of male and female venoms were also observed, indicating a possible intraspecific variation in the composition of the venom of this Brazilian *Sicarius* sp. spider, as we have previously described for *Loxosceles* venoms [33], [34]. Interestingly, the major component in all tested venoms had a Mr of 32–35 kDa, which corresponds in *Loxosceles* venoms to the main toxic components, the SMases D. This major band, in the *Sicarius ornatus* spider venoms, was also recognized by a monospecific polyclonal serum elicited against *Loxosceles* SMases D, confirming the presence of SMase D related proteins in male and female venoms from *Sicarius ornatus*.

It has been shown that the SMase D activity from African *Sicarius* venoms was similar to that of *Loxosceles* from the Americas; however, little or no activity, at the same venom concentrations, was detected in samples from several South and Central American *Sicarius* species [16]. Nevertheless, our results showed here indicate that venoms of a Brazilian *Sicarius*, *S. ornatus*, is able to hydrolyze sphingomyelin and that the enzymatic activity was similar to that determined for *Loxosceles* venoms. However, the SMase D activity of *Sicarius ornatus* male venom was statistically lower than female and *Loxosceles* spp. venoms, which reinforce the idea of intraspecific variation in the composition and toxicity of the Brazilian *Sicarius ornatus* venoms. The discrepancy between our data and results from the study of Binford and collaborators [16] are most likely due to interspecies variations of South American *Sicarius* venoms. Besides, it was not mentioned if the New World *Sicarius* venoms tested were collected from male or female spiders [16], thus it is possible to consider that the low SMase D activity detected may be also due to the presence of high amounts of male venoms, with lower activity, in the samples used in the experiments.

The venom of *Sicarius albospinosus* from South Africa can induce systemic effects, including disseminated intravascular coagulation in rabbits, but the same effect was not observed with *Sicarius testaceus* (South Africa) venom [5], [9]. Thus, these data also suggest that interspecific variations in the venom composition may contribute to the severity of the *Sicarius* envenomation. Although some studies have investigated the expression and the SMase D activity in *Sicarius* spider venoms, none of them has addressed the important clinical manifestation induced by *Loxosceles* SMases D, namely, hemolysis.

Investigations focusing on the effects of *Loxosceles* venoms on erythrocytes demonstrated that SMase D induced activation of membrane bound metalloproteinases, resulting in cleavage of glycophorins, which facilitated activation of complement *via* the alternative pathway resulting in lysis of the cells [22]. In order to assess whether the *Sicarius ornatus* could also induce Complement-dependent hemolysis, erythrocytes were incubated with male or female crude venoms. Both venoms were able to render human erythrocytes susceptible to lysis by autologous complement, although with less potency than *Loxosceles* spp. venoms. Moreover, as with *Loxosceles* spp. venoms, male and female *Sicarius ornatus* venoms were able to significantly reduce the binding of anti-GPC antibodies, indicating the recognition of extracellular epitopes close to the membrane. Again, the female venom was more active than male venom.

The ability of the *Loxosceles* SMase D to bind to different species of erythrocytes (such as human, sheep, rats, rabbits and guinea pigs) as well as to several cell types (such as epidermal cells, hepatocytes, monocytes, B and T cells, endothelial cells, platelets and neutrophils) have been already described [15], [35]. Although no specific receptor has been described yet for this interaction, the SMase D binding ability certainly is an important step for the mechanism of action of the *Loxosceles* spider venoms. As shown here, the reduction of GPs epitopes, induced by both *Sicarius ornatus* and *Loxosceles* venoms, correlated with the binding of the SMases D to the erythrocyte cell surface as detected by anti-SMase D serum. Although, the data obtained also suggest that the SMases D from *Loxosceles* can bind to the cells membrane with higher efficiency than *Sicarius ornatus* ones, but this may be in part a result of differences in the antigenic recognition, since the anti-SMase D serum used was produced against a pool of native *Loxosceles* SMases D. Together, these observations indicate that *Sicarius ornatus* venoms also have the ability to induce complement-dependent hemolysis, which may occur by the same hemolytic molecular mechanism displayed by *Loxosceles* venoms.

Several reports have shown that *Sicarius* as well as *Loxosceles* venoms are able to cause dermonecrotic lesions in humans and in experimental animals [3], [9], [21], [23], [25], [26], [34]. We have previously demonstrated that *Loxosceles* spider venom induces an increase in cell death in the keratinocytic human cell line HaCaT [26]. Here, when the HaCaT cells were incubated with *Loxosceles* or *Sicarius ornatus* female venoms, a significant decrease in the cell viability was observed, suggesting that *Sicarius ornatus* venoms may also share similar molecular mechanisms leading to tissue damage and development of dermonecrosis with the *Loxosceles* venoms. After 72 h, *Sicarius ornatus* male venom has only induced a small reduction in the cell viability, data that once more strengthens the idea of intraspecific variation in *Sicarius ornatus* venom toxicity. The lower cytotoxic effect exhibited by male venom did not correlate well to its significant sphingomyelinase activity. The method used for measuring and comparing the sphingomyelinase activity of the venoms is based on the hydrolysis of the substrate sphingomyelin dispersed in a buffer, which maybe does not reflect the *in vivo* lipase activity on real substrates, *e.g.*, sphingomyelin present on intact cell membranes.

Finally, if the amount of protein and volume of the venom in the poisonous gland is taken into account, the toxic potential of *Sicarius ornatus* bite is greater than that of *Loxosceles*, since *Sicarius ornatus* contains higher volume (data not shown) and higher amount of protein in its venom gland than *L. laeta* spider, whose venom contains the highest protein concentration in *Loxosceles* species of medical importance in Brazil [33], [34]. Yet, the reason why few incidences of envenomation by *Sicarius* in Brazil are reported may lie on the differences in habitat and on the low exposure to humans by the *Sicarius* species.

In conclusion, we show here, for the first time, that a Brazilian *Sicarius* spider species, *Sicarius ornatus*, is able to cause haemolysis and keratinocyte cell death similar to the South American *Loxosceles* species, harmful effects that were positively associated with the presence of active SMases D and with *in vivo* pathologies. Therefore the venom of *S. ornatus* has the potential to cause serious pathology upon envenomation, similar to that observed after *Loxosceles* envenomation. Our results also suggest that, in addition to the interspecific differences, intraspecific variations in the venoms composition may play a role in the toxic potential of the New World *Sicarius* venoms species.
